# Small mobile sequences in bacteria display diverse structure/function motifs

**DOI:** 10.1111/j.1365-2958.2007.06068.x

**Published:** 2007-12-12

**Authors:** Nicholas Delihas

**Affiliations:** Department of Molecular Genetics and Microbiology, School of Medicine SUNY, Stony Brook, NY 11794-5222, USA

## Abstract

Small repeat sequences in bacterial genomes, which represent non-autonomous mobile elements, have close similarities to archaeon and eukaryotic miniature inverted repeat transposable elements. These repeat elements are found in both intergenic and intragenic chromosomal regions, and contain an array of diverse motifs. These can include DNA sequences containing an integration host factor binding site and a proposed DNA methyltransferase recognition site, transcribed RNA secondary structural motifs, which are involved in mRNA regulation, and translated open reading frames found fused to other open reading frames. Some bacterial mobile element fusions are in evolutionarily conserved protein and RNA genes. Others might represent or lead to creation of new protein genes. Here we review the remarkable properties of these small bacterial mobile elements in the context of possible beneficial roles resulting from random insertions into the genome.

## Introduction

A class of small DNA repetitive sequences, first discovered in *Neisseria* sp. (Correia *et al*., 1988), are present in genomes of diverse bacteria. These fascinating elements are continuously being discovered as more genomes are sequenced. Repeat elements represent non-autonomous mobile units of < 200 bp, which do not encode proteins. Most carry terminal inverted repeat sequences (TIR), have a ^5′^TA^3′^ dinucleotide motif at their terminal ends, are A + T-rich and produce target site duplications. These bacterial elements closely resemble the archaeon and eukaryotic miniature inverted repeat transposable elements (MITE; Redder *et al*., 2001; Feschotte *et al*., 2002) in terms of the above-mentioned molecular properties. Transposases are proposed to mobilize archaeon and eukaryotic MITEs based on similarities of TIR sequences and the terminal sequences of transposons. Furthermore, the interaction of a transposase with a eukaryote MITE has been demonstrated via *in vitro* binding studies (Feschotte *et al*., 2005). With the bacterial repeat elements, similarities between TIR and IS transposon sequences have either not been looked for or not been found, with the exception of the repeat unit of pneumococcus (RUP) in *Streptococcus pneumoniae*, which is proposed to be mobilized by a transposase of the insertion sequence IS*630*-Spn*1* (Oggioni and Claverys, 1999). Several bacterial repeat sequences have previously been referred to as MITEs (De Gregorio *et al*., 2006; Siguier *et al*., 2006). In this review, the same terminology will also be used in view of the similarities of the bacterial elements with archaeon and eukaryotic MITEs. Other classes of bacterial repeat sequences, such as bacterial interspersed mosaic elements (Bachellier *et al*., 1999; Espeli *et al*., 2001), represent separate families of repeat sequences and have interesting properties of their own, but will not be covered here.

Bacterial MITE sequences are primarily found in intergenic regions of the chromosome, but they are also present intragenically (Ogata *et al*., 2000; 2005; Delihas, 2007). Many bacterial MITEs carry contiguous open reading frames that are characteristically found fused, in frame to other open reading frames. Here we review properties and proposed functions of these non-autonomous small mobile elements in the context of Jacob's (1977) intuitive concept of biological ‘tinkering’, i.e. random insertion of these small sequences into the genome, whereby fusion of two or more units can lead to new functions.

## Properties of repeat elements

Structural properties of bacterial MITEs are summarized in Table 1. In addition to properties listed, repeat units of all families are small (∼100–200 bp), and their transcripts display highly base-paired stable secondary structures consisting of long stem loops.

**Table 1:** Properties of repeat elements.

Acronym	Organism	TIR	TIR size^a^	TSD	^5′^TA^3′^ at termini	DNA motif	Open reading frame
Correia/NEMIS	*Neisseria* sp.	+	24 bp	+	+	+/−b	+/−c
ERIC/IRU/RU-1	*E. coli* and other enterobacteria	+	7 bp	+	+	−	+
RUP	*S. pneumoniae*	+	9 bp	+	+	−	−
RPE	*Rickettsia* sp.	+	12 bp	?	+/−d	−	+
CIR	*Caulobacter* and *Brucella* sp.	−e	−	?	+	+	−
YPAL/RU-2	*Yersinia* sp. and *Erwinia carotovora*	–	7 bp^f^	+	–	–	+
RU-3	*E. coli* and other enterobacteria	+	4 bp	?	+/−g	–	+

a Indicates only number of contiguous Watson/Crick base pairs. Stem loops are larger with A-G, U-U, etc. mis-pairing in stem structures.

b 155 bp species has a DNA motif, the 107 bp species does not (Buisine *et al*., 2002).

c 107 bp species has open reading frames (*Supplementary material*). The 155 bp species does not have a contiguous open reading frame.

d Not all RPE elements have a ^5′^TA^3′^motif.

e The 5′ and 3′ sections of RNA model independently form long stem loops (Chen and Shapiro, 2003).

f Internal bp closest to the 5′-3′ ends.

g The 3′ terminal end lacks the ^5′^TA^3′^ motif (Delihas, 2007).

TSD, target site duplication.

**Fig. 1:**
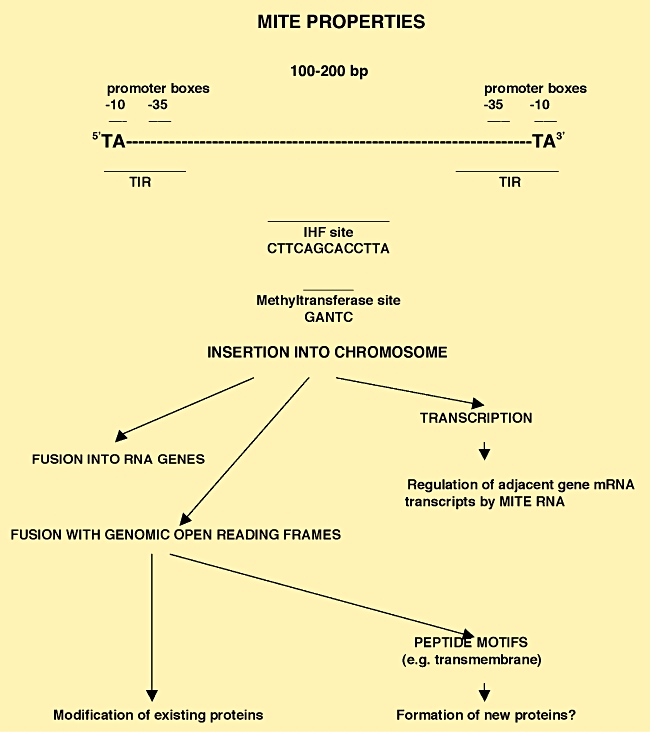
Diagrammatic representation of bacterial MITE properties. Top line drawing depicts one strand of the MITE DNA with various motifs also shown.

Bacterial MITEs display eclectic properties because of information embedded at different levels, i.e. the DNA sequence, and the transcribed and translated sequences (Fig. 1). At the DNA sequence level, there are TIR that are characteristic of IS elements and transposition (Oggioni and Claverys, 1999; Mazzone *et al*., 2001; Buisine *et al*., 2002), putative promoter sites (Buisine *et al*., 2002), an integration host factor (IHF) binding site (Buisine *et al*., 2002), and a putative DNA methyltransferase binding site (Chen and Shapiro, 2003). At the transcribed RNA level, MITE RNA secondary structures participate in regulation of mRNA transcripts of adjacent genes (Mazzone *et al*., 2001; De Gregorio *et al*., 2005). Many MITEs display open reading frames. At the translated peptide level, MITE amino acid sequences are found fused in frame to open reading frames in protein-encoding genes (Ogata *et al*., 2000; Abergel *et al*., 2006; Delihas, 2007). Some open reading frame fusions contain protein structural motifs, such as predicted transmembrane domains and/or signal peptide sequences, and these fusions might lead to the creation of new proteins (Delihas, 2007). Yet, adding another dimension to mobile element fusions, repeat sequences have been found inserted into RNA genes (Ogata *et al*., 2002). Although archaeon and eukaryotic MITEs have not been reported to display functional motifs at the DNA level, some archaeon repetitive elements also have open reading frames, and these repeat DNA sequences are found fused into protein genes (Suyama *et al*., 2005).

## Motifs in MITE sequences

### Correia/*Neisseria* miniature insertion sequences

*Neisseria* repeat sequences consist of four related elements (Buisine *et al*., 2002) and are termed Correia or *Neisseria* miniature insertion sequences (NEMIS; Correia *et al*., 1988; Mazzone *et al*., 2001; Buisine *et al*., 2002; Liu *et al*., 2002). Two of these elements are ∼107 bp and ∼155 bp. They are similar in sequence except that the 155 bp element has a 50 bp internal sequence. An alignment of filled and empty genomic sites can provide a repeat sequence size, e.g. alignment of sites with and without the *Neisseria* sp. 107 bp repeat sequence shows that the empty site is 105 bp, which suggests that the putative mobile sequence is 105 bp (Fig. S1).

Buisine *et al*. (2002) showed that the 50 bp internal sequence of the 155 bp species carries an IHF binding site. An analysis of gel mobility shifts shows that this site binds IHF *in vitro*. In addition, putative divergent promoters were located within the sequence of the element (Buisine *et al*., 2002; Fig. 1). Functions of the IHF site and putative promoter sequences are unclear, but they might modulate expression, and/or the IHF might facilitate transposition of the 155 bp repeat element (Buisine *et al*., 2002).

At the transcribed RNA level, the Neisserial MITE RNA has been proposed to participate in regulation of adjacent gene mRNA transcripts by the double-stranded cleaving enzyme RNase III, via NEMIS RNA transcript stem-loop secondary structures (Mazzone *et al*., 2001; De Gregorio *et al*., 2002; 2003). At the translated amino acid level, the 107 bp species has two open reading frames that are found fused to numerous other open reading frames, including those of three different classes of transposase genes (Tables S1 and S2, Fig. S1). The 155 bp repeat displays multiple stop codons in all reading frames and, with one exception, is not found inserted into open reading frames. Thus, the 107 bp species has open reading frames that are abundantly found fused in intragenic regions. The 155 bp species does not have contiguous open reading frames, but has an IHF motif and is almost exclusively found in intergenic regions. Accordingly, these two related MITE species appear to vary in locations in the genome vis-à-vis intragenic/intergenic regions.

### *Caulobacter* CerM-associated intergenic repeat sequences

Cell cycle-regulated methyltransferase (CerM), a DNA methyltransferase enzyme in *Caulobacter*, *Brucella* and other alpha proteobacteria, regulates gene transcription and timing of DNA replication initiation (Berdis *et al*., 1998; Chen and Shapiro, 2003). CerM methylates the A residue in the nucleotide sequence GANTC. This recognition site is also found in intergenic repeat elements, termed *Caulobacter* CerM-associated intergenic repeat (CIR) sequences. These repeats are abundant in *Caulobacter* and related alpha proteobacteria genomes. CIR elements are highly conserved between alpha proteobacteria species. Four species of the ∼110 bp repeat sequences, termed CIR1–CIR4, have copies of the GANTC methyltransferase recognition motif, and this sequence within the repeat unit is also highly conserved phylogenetically. A functional role for the CIR-associated GANTC motif has not been determined, but Chen and Shapiro (2003) speculate that it may be related to regulatory functions of CerM.

Proposed RNA secondary structures of CIRs from *Caulobacter* and *Brucella* reveal phylogenetically conserved properties (Chen and Shapiro, 2003). CIR RNA secondary structures show two long inverted repeats which form two arms and these are connected by a linker sequence. The GANTC motif is at or partly encompasses a terminal loop of one of the RNA stem loops, and therefore is highly exposed. Secondary structures of CIR RNAs differ from those of other bacterial repeat elements, which have no arms or linker sequences, but contain a stem loop formed by 5′-end-3′-end base-pairing. Perhaps the CIR structures evolved to have the GANTC motif exposed. CIR sequences have not been found intragenically, and all six open reading frames have multiple stop codons (N. Delihas, unpublished). CIR elements only partially resemble MITE characteristics and more information is needed to ascertain the mechanism of mobility.

### Repeat unit of pneumococcus

The RUP is 107 bp in length and is found repeated 108 times intergenically in *S. pneumoniae* (Oggioni and Claverys, 1999). Indirect evidence suggests that the RUP is mobilized by a transposase encoded by the insertion sequence IS*630*-Spn*1*. This is based on the high degree of sequence similarity between the RUP TIR sequences and those of IS*630*-Spn*1*, as well as insertions at target sites that contain the TA dinucleotide. Interestingly, RUP appears to target genomic regions that have IS elements, and more than six IS families have been reported to have RUP insertions, which lead to inactivation of the IS transposase genes (Oggioni and Claverys, 1999). This raises the question of whether the pneumococcus MITE functions as a regulator to limit the number of transposase genes in the cell. MITE insertion into transposase genes of *Neisseria* has also been observed (Table S2), and therefore the same question applies.

Functional motifs at the DNA sequence level have not been reported for RUPs. In addition, the RUP sequence has stop codons in all six different reading frames. Thus, despite providing the best evidence for the function of MITE TIRs in transposition, the RUP element thus far shows no other structure/function signatures. The RUP is believed to retain mobility, and the high sequence conservation of repeats in the genome might indicate that insertions took place in recent evolutionary time (Oggioni and Claverys, 1999).

### *Rickettsia*-specific palindromic elements

*Rickettsia* sp. contain 105–146 bp termed *Rickettsia*-specific palindromic element (RPE; Ogata *et al*., 2000; Claverie and Ogata, 2003). Secondary structural modelling of the RPE sequences predicts long stem-loop structures with terminal inverted repeats (Ogata *et al*., 2000). The sequence shown by Ogata *et al*. (2000) has no ^5′^TA^3′^ dinucleotide motif, but at least one other RPE species has the ^5′^TA^3′^ motif at both terminal ends (e.g. the RPE at nucleotide positions 70591–70736 of *Rickettsia conorii* str. Malish 7).

In addition to their presence in intergenic regions, the unprecedented work of Claverie and coinvestigators shows that RPE sequences have open reading frames that are found fused in frame in *Rickettsia* protein genes (Ogata *et al*., 2000). Intragenic insertions were observed in 19 reading frames of protein genes, many of which encode phylogenetically conserved proteins. Experimental analyses of one gene product containing the insert sequence, the guanylate kinase encoded by *gmk*, shows that this gene is translated, and the gene product displays enzymatic activity, although the substrate affinity was changed relative the RPE-minus control. Thus, the RPE insert might have a neutral or slightly detrimental effect on enzyme function (Abergel *et al*., 2006). The *gmk* intragenic fusion can be looked at as a random event that resulted in a neutral effect on protein function, and therefore is tolerated by the cell.

Of major interest also is the finding of RPE elements fused in two Rickettsial RNA genes, which encode tmRNA and the ribozyme M1 RNA (Ogata *et al*., 2002). The repeat element inserts in these RNA genes may have a neutral effect on function, as was found with some protein gene fusions. However, the finding of RNA gene fusions expands the types of genes where repeat element fusions are found.

### Enterobacterial repetitive intergenic consensus/IRU/RU-1

Two groups independently discovered 127 bp repeat sequences in enterobacteria, termed enterobacterial repetitive intergenic consensus (ERIC)/IRU/RU-1 (Sharples and Lloyd, 1990; Hulton *et al*., 1991). These repeat elements are well characterized (De Gregorio *et al*., 2005; Wilson and Sharp, 2006; Delihas, 2007).

The repeat elements appear to be involved in regulation of mRNA stability. *In vivo* studies with *Yersinia* sp. show ERIC sequences co-transcribed with upstream and downstream genes, and that upstream gene transcripts are less stable than the downstream transcripts (De Gregorio *et al*., 2005). De Gregorio *et al*. (2005) make a good case for destabilization of upstream transcripts via ERIC RNA conformational change and ribosome-induced exposure of an RNase E cleavage site. It should be pointed out that the orientation of the ERIC sequence is crucial for this effect (De Gregorio *et al*., 2005). Their work represents the best evidence so far for a functional role of MITEs in the cell, and their findings appears to follow Jacob's (1977) concept of biological ‘tinkering’, i.e. random insertion of sequences into the genome, whereby fusion of two or more units can lead to new functions.

DNA sequence structure/function motifs other than TIRs have not been found. On the other hand, the 127 bp MITE has contiguous open reading frames that are found fused in frame intragenically (Delihas, 2007). Fusions are in several conserved protein genes, but also, fusions of a MITE frame shift sequence result in formation of new gene loci encoding putative membrane proteins with predicted transmembrane domains. One particularly interesting fusion in *Salmonella*, a putative inner membrane protein at gene locus *STM0083* of *Salmonella* Typhimurium LT2, is phylogenetically conserved in five species of *Salmonella*.

### *Yersinia* palindromic sequence/RU-2

A repeat element of ∼168 bp, termed *Yersinia* palindromic (YAPL) sequence or RU-2, is abundantly found intergenically in *Yersinia* sp. (De Gregorio *et al*., 2006; Delihas, 2007). The 168 bp mobile element differs from other bacterial repeat elements. The termini do not have the ^5′^TA^3′^ dinucleotide motif, the inverted repeats are internal and the repeat element is not A + T-rich. YPAL/RU-2 elements do induce target site duplications upon insertion to the genome, but these vary in size from 3 to 26 bp (De Gregorio *et al*., 2006). Genomic target sequences either span or are next to areas of dyad symmetry. YPAL elements are predicted to be recently mobile in evolutionary time based on comparisons of empty and filled chromosomal sites in *Yersinia pseudotuberculosis* and *Yersinia pestis*. These elements are also co-translated with upstream genes and might influence RNA the stability of upstream transcripts (De Gregorio *et al*., 2006).

As with several other bacterial repeats, the *Yersinia* 168 bp repeat sequence displays a contiguous open reading frame (Delihas, 2007). Of major interest is that the translated sequence, which comprises 56 amino acids, contains a high percentage of hydrophobic residues, particularly in one major cluster. Fusion of the mobile element in frame with other open reading frames was detected. In some cases, these fusions formed genes encoding putative proteins that display a predicted signal peptide motif. One particularly interesting locus, *YPO1552*, encodes a 144-amino-acid putative exported protein with a signal peptide partially formed by the MITE sequence. This gene locus is 100% conserved in *Y. pestis* and *Y. pseudotuberculosis*. It has been hypothesized that this *Yersinia* open reading frame represents a new exported protein resulting from fusion of the mobile element with a chromosomal segment whose translated sequence displays a predicted signal peptide motif (Delihas, 2007).

### RU-3

RU-3 is a 103 bp repeat unit found intergenically in *Escherichia coli* and *Shigella* sp. (Delihas, 2007). The element displays a long stable stem-loop secondary structure but imperfect inverted repeats and a ^5′^TA^3′^ motif only at the 5′ end. As the RU-3 sequences are only partially conserved, this element might represent an evolutionarily older MITE sequence and is probably no longer mobile.

The RU-3 sequence has translatable open reading frames, and fusions are found mostly in hypothetical proteins. Of interest, however, is the putative LivJ protein, which represents a fusion of motifs consisting of part of an acetyl-transferase sequence at the amino terminal end, a spacer sequence and the RU-3 at the C-terminal end. Fusion of other MITEs with sequences that carry conserved functional domains has been observed in several cases (Delihas, 2007). These domain fusions are consistent with Jacob's (1977) tinkering hypothesis, whereby random combinations of two units (domains) create a more elaborate one.

## Conclusions and future prospects

Bacterial repeat elements are rich in motifs present at the DNA, RNA and translated peptide levels. How did these motifs originate? TIRs might have evolved from IS sequences, as the RUP TIR comparisons indirectly suggest. Did the *Neisseria* NEMIS IHF sequence, the *Caulobacter* CIR methyltransferase recognition site or sequences representing peptide motifs originate by a capture of domains from existing chromosomal sequences? Or evolve within repeat sequences by random base-pair changes? The mobile elements are hypothesized to move via transposases, but there is yet no experimental support for this. How these mobile sequences are transposased within the chromosome is a relevant question for future work.

Miniature inverted repeat transposable element insertions appear to be biased random events (biased because of target site recognition), but key insertions can result in new functional roles, e.g. inserts in downstream regions of genes and subsequent participation of the MITE RNA transcripts in mRNA regulation. There is ample evidence that putative gene loci are created with MITE open reading frame fusions, but these need to be tested experimentally to show the existence of a translation product. A putative inner membrane protein gene (locus *STM0083*) originating from a MITE fusion in *Salmonella* offers a hint of functionality, as it shows phylogenetic conservation. The predicted exported protein gene (locus *YPO1552*) also stands out as an interesting construct that partly originated from a mobile element. Fusion of a mobile unit into a conserved protein gene, where addition of the mobile sequence improves enzyme function, has not been shown, but a large number of Rickettsial genes that contain RPE inserts have not yet been analysed. Thus, one cannot exclude a positive effect of an insertion on enzyme function. The finding of RPE sequences in RNA genes adds to the possible evolutionary significance of repeat element fusions. In eukaryotes, a family of primate microRNA genes was shown to be derived from a MITE sequence (Piriyapongsa and Jordan, 2007). This important new finding adds another dimension to the significance of MITE genomic fusions. The prospect of bacterial regulatory RNA genes arising from repeat element fusions should also be looked into in future studies.

Prominent among the motifs are the CIR methyltransferase recognition sequence, as it is phylogenetically conserved, and the IHF sequence, which is functional *in vitro*. MITEs carrying these motifs might constitute reservoirs, where upon further genetic rearrangement, can function in newly formed chromosomal loci. Could this have occurred in the past? To investigate this, sequences upstream of genes can be scanned for ‘remnants’, for example, sequences that are partly similar to the *Neisseria* 155 bp sequence but still carry the IHF motif. A phylogenetic consistency in presence of the repeat sequence in similar upstream regions of orthologous genes, e.g. in *Neisseria meningitidis* and *Neisseria gonorrhoeae*, would support the notion of a putative past event in an ancestral organism.

In summary, bacterial mobile sequences appear to be important vehicles for evolutionary change and, as more are discovered, we may have a better insight into roles of these versatile and multifaceted elements.

## Supplemental material

This material is available as part of the online article from:

http://www.blackwell-synergy.com/doi/abs/10.1111/j.1365-2958.2007.06068.x

(This link will take you to the article abstract).
