# Supplementary material for: Small mobile sequences in bacteria display diverse structure/function motifs
Source: Mol Microbiol. 2007 Dec 12;67(3):475–81. doi: 10.1111/j.1365-2958.2007.06068.x (PMC2229807; doi:10.1111/j.1365-2958.2007.06068.x)
Supplement: Supplementary file 1 [file mmi0067-0475-SD1.pdf]

Small mobile sequences in bacteria display diverse structure/function motifs

Supplement

Nicholas Delihas, Department of Molecular Genetics and Microbiology, School of Medicine, SUNY, Stony Brook, NY 11794-5222 USA

Neisseria meningitidis Correia/NEMIS repeat element

A 107 bp repeat element is found in *Neisseria* sp. in both intergenic and intragenic regions of chromosomes. The nucleotide sequence of the element, located at positions 933983-933877 in the *N. meningitidis FAM18* genomic sequence is:

5'TATAGTGGATTAAATTTAAACCAGTACAGCGTTGCCTCGCCTTGCCGTACT
ATTTGTACTGTCTGCGGCTTCGTCGCCTTGTCCTGATTTAAATTTAATCCACT
ATA3'

This sequence displays a contiguous open reading frame in the frame +1 and a partial open reading frame in the frame -1:

$_{1}$YSGLNLNQDKATKPQTVQIVRQGEATLYWFKFNPL$_{35}$

$_{1}$YSGLNLNQYSVASPCRTICTVCGFVALS$_{28}$

The 107 bp sequence is found fused to gene loci that encode transposase and other annotated gene loci. Table S1 shows sixteen gene loci where the 107 bp sequence open reading frames (predominantly the frame-1) are fused to other open reading frames. These loci were identified with the NCBI Blast program using a protein blastx search (web site http://www.ncbi.nlm.nih.gov/BLAST/). E - values are as shown by the blast program. The loci shown in Table S1 are from

the following species: *N. meningitidis MC58, N. meningitidis Z2491, N. meningitidis FAM18* and *N. gonorrhoeae FA 1090*.

Table S2 displays transposase gene loci from three different families of transposases that contain the 107 bp repeat element. Positions of repeat sequence within transposase sequences are also shown. Only the frame +1 amino acid sequence is found fused to the transposase open reading frames.

A comparison of sequences from transposase gene loci *NMB0991* and *NMB1539* in *N. meningitidis MC58*, as well as the sequence from the orthologous transposase locus *NMA0458* from *N. meningitidis Z2491* is in Fig. S1. The alignment shows the 107 bp insert sequence is in *NMB0991* and *NMA0458* but not in *NMB1539*. The sequence alignment suggests that during fusion, a $^{5'}$TA$^{3'}$ dinucleotide was duplicated and there was no replacement of sequences in the target site but an addition of the mobile sequence. The alignment also suggests that the mobile sequence is 105-bp.

Table S1
Gene loci containing *N. meningitidis* 107 bp insert

Locus	Proposed protein	aa chain length	insertion site	%identity	Score	Expect value
NMB1858	hypothetical protein	128 aa	(3-32)	100%	65.9	1e-12
NMC0306[a]	putative integral membrane protein	135 aa	(108-135)	96%	60.8	3e-11
NMB1917[a]	hypothetical protein	135 aa	(108-135)	96%	60.8	3e-11
NMB2149	hypothetical protein	86 aa	(59-86)	96%	89.3	3e-11
NMB0676[b]	hypothetical protein	133 aa	(106-133)	96%	60.8	3e-11
NMA0876[b]	hypothetical protein	133 aa	(106-133)	96%	60.8	3e-11
NGO1359	hypothetical protein	83 aa	(18-40)	91%	49.3	2e-10
NGO0015	hypothetical protein	94 aa	(46-68)	86%	47.8	3e-10
NMB1695	hypothetical protein	326 aa	(299-326)	86%	37.4	4e-04
NGO1034	hypothetical protein	114 aa	(25-52)	88%	36.6	6e-04
NMB1837	hypothetical protein	84 aa	(64-84)	94%	36.2	8e-04
NGO1604	hypothetical protein	80aa	(18-35)	72%	33.5	0.005
NGO1255	hypothetical protein	113 aa	(96-113)	94%	32.7	0.009
NMA0537[a]	predicted membrane protein	121 aa	(108-121)	92%	30.8	0.035
NMA1071	hypothetical protein	139 aa	(126-139)	92%	30.8	0.035
NMB1915	hypothetical protein	129aa	(79-89)	100%	23.9	3.7

[a] loci are orthologs; [b] loci are orthologs

Table S2

Neisseria meningitidis **transposase genes with 107 bp insert sequence**

Transposase locus	aa chain length	aa positions of insert
NMB0991 IS1106 transposase_11 IS5 family	370 aa	283-317
NMA0458 IS1106 transposase_11 IS5 family	370 aa	283-317
NMA1641[a] IS1106 transposase_11 IS5 family	273 aa	186-220
NMC1340[b] IS1106 transposase_11 IS5 family	166 aa	79-113
NMB1386 has transposase_14 IS630 family	151 aa	14-48
NMC1006 has Tra5/integrase core domain	249 aa	189-223

[a]missing first 97 aa at N-terminal
[b]missing first 204aa at N-terminal

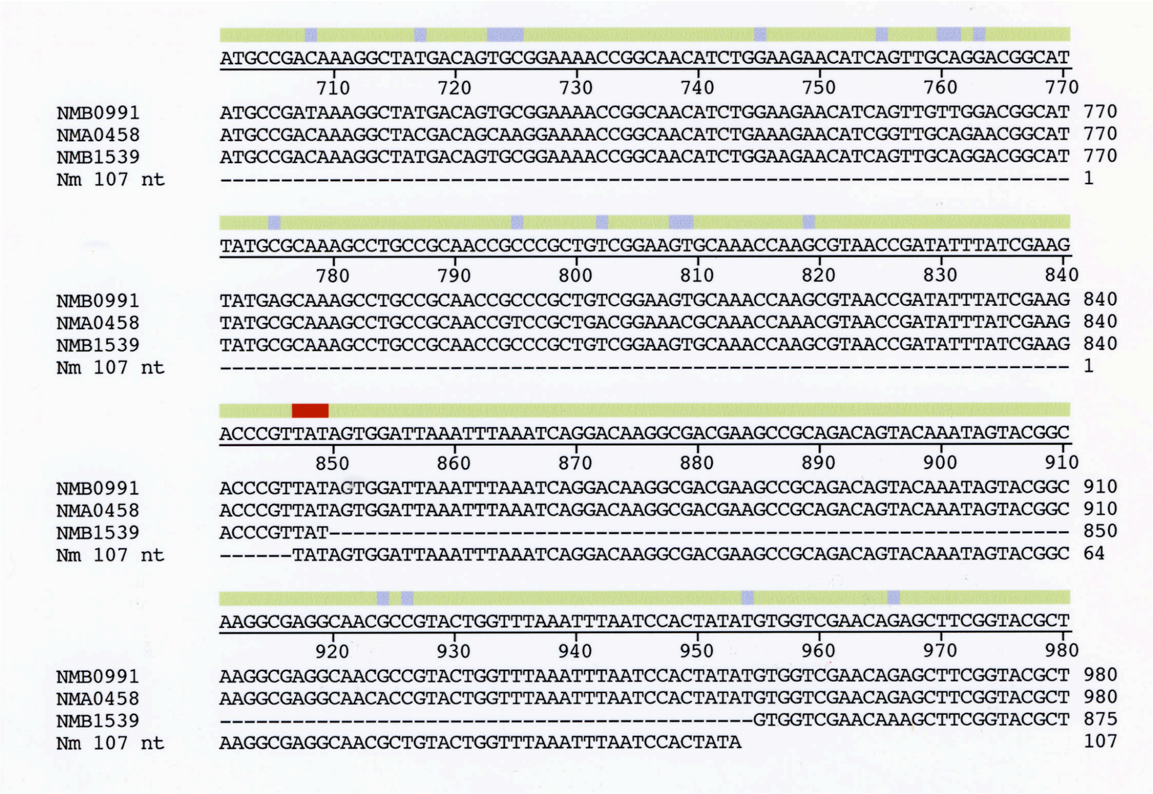

Fig. S1. Alignment of nucleotide sequences from the 5' ends of transposase gene loci *NMB0991* and *NMB1539* from *N. meningitidis MC58*, the orthologous transposase locus *NMA0458* from *N. meningitidis Z2491*, and the 107 nt MITE unit sequence from *N. meningitidis FAM18*. Alignment was by the DNASTAR, Inc program using the ClustalW alignment program. Note that the alignment of T at position 849 of *NMB1539*, as produced by the alignment program, is arbitrary. However alignment of this T at position 954 is more consistent, as it shows no gap with the Nm 107 nt sequence.
